# P-170. Efficacy and Safety of Intermittent Preventive Treatment with Dihydroartemisinin-Piperaquine for Prevention of Malaria in Pregnant Women with HIV: A Meta-analysis of Randomized Controlled Trials

**DOI:** 10.1093/ofid/ofae631.375

**Published:** 2025-01-29

**Authors:** Meghna Joseph, Mrinal Murali Krishna, Chidubem Ezenna, Lal Sadasivan Sreemathy

**Affiliations:** Medical College Thiruvananthapuram, Thodupuzha, Kerala, India; Medical College Thiruvananthapuram, Thodupuzha, Kerala, India; UMass-Baystate medical center, Springfield, Massachusetts; ReAct Asia-Pacific, Global Institute of Public Health, Thiruvananthapuram, Kerala, India

## Abstract

**Background:**

WHO recommends intermittent preventive treatment in pregnancy (IPTp) with sulfadoxine-pyrimethamine (S-P) in malaria-endemic areas. In pregnant women with HIV taking cotrimoxazole prophylaxis, IPTp with S-P is contraindicated due to the risk of adverse effects from combining two antifolate and sulfonamide drugs. The growing resistance of the malarial parasite to sulfa-based drugs adds to the problem. We performed a meta-analysis comparing IPTp with dihydroartemisinin-piperaquine (D-P) vs. placebo for malaria prevention in pregnant women with HIV.

**Figure:**
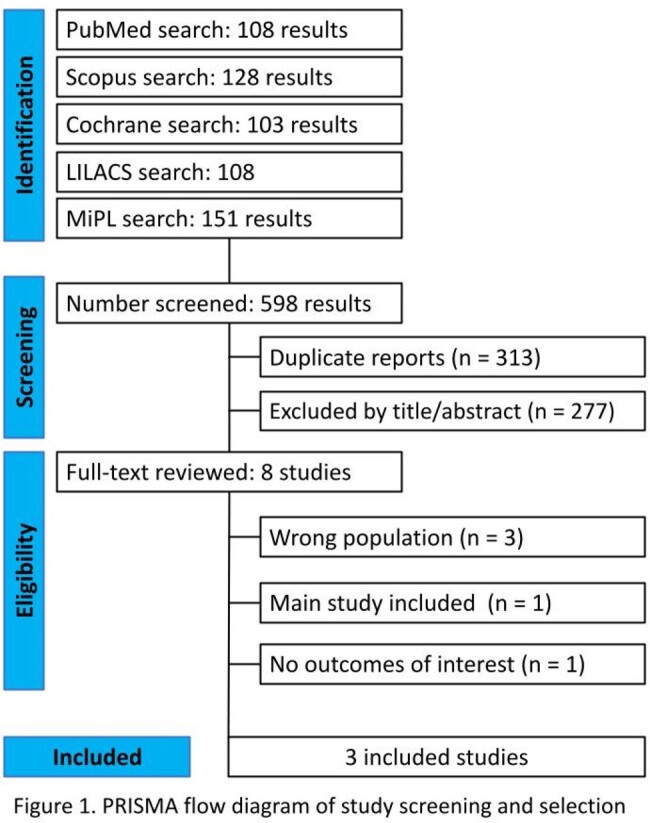

**Methods:**

We searched PubMed, Scopus, Cochrane Central, LILACS, and Malaria in Pregnancy Consortium Library databases for trials comparing IPTp with D-P vs. placebo in pregnant women with HIV. Outcomes of interest included placental malaria, maternal malaria, and adverse pregnancy outcomes. Statistical analysis was performed using R software. Heterogeneity was assessed using I^2^ statistics. The analysis was conducted following the Preferred Reporting Items for Systematic Reviews and Meta-Analysis guideline.

**Figure:**
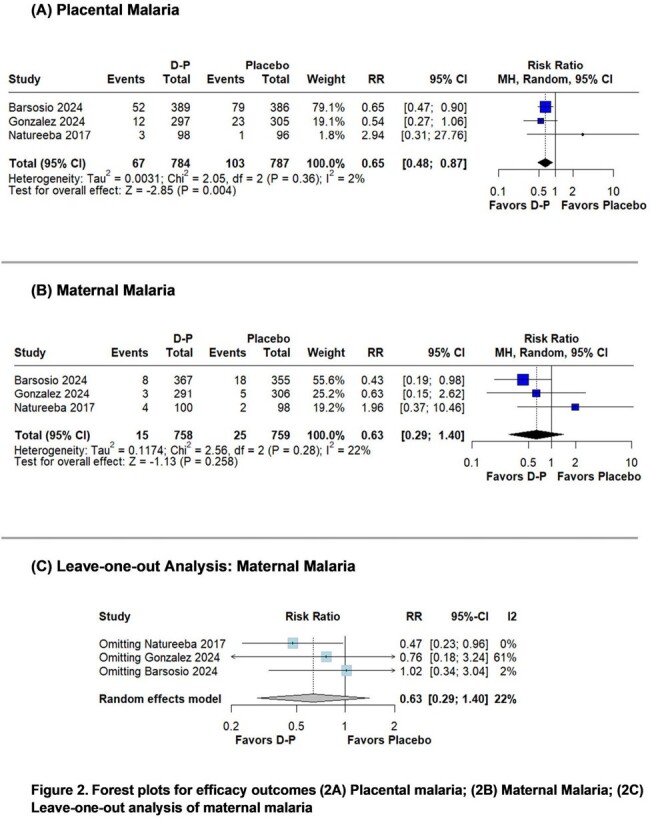

**Results:**

The systematic review identified 3 randomized controlled trials including 1,770 participants. Placental malaria (RR 0.65; 95%CI 0.48-0.87; p=0.004; I^2^=2%) at delivery was significantly lower in the D-P group compared with the placebo group. Maternal malaria (RR 0.63; 95%CI 0.29-1.40; p=0.258; I^2^=22%) at delivery showed no significant difference between the groups. However, a leave-one-out analysis excluding one study conducted during a low-transmission period found a significantly lower risk of maternal malaria (RR 0.47; 95%CI 0.23-0.96; p=0.039; I^2^=0%) in the D-P group. Adverse pregnancy outcomes like mother-to-child transmission of HIV (RR 1.53; 95%CI 0.25-9.35; p=0.646; I^2^=0%), spontaneous abortion (RR 1.80; 95%CI 0.60-5.42; p=0.296; I^2^=0%), stillbirth (RR 1.02; 95%CI 0.56-1.85; p=0.952; I^2^=0%), congenital anomaly (RR 0.90; 95%CI 0.33-2.43; p=0.830; I^2^=31%), preterm delivery (RR 1.05; 95%CI 0.56-1.94; p=0.885; I^2^=42%), and low birth weight (RR 1.13; 95%CI 0.87-1.48; p=0.352; I^2^=0%) were similar in both groups.

**Figure:**
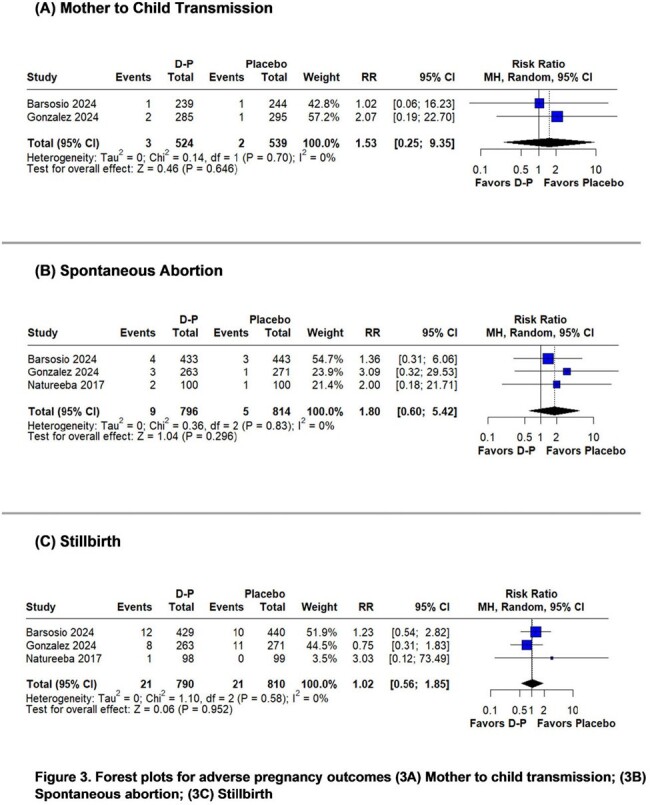

**Conclusion:**

IPTp with D-P reduced the risk of placental malaria and maternal malaria at delivery in pregnant women with HIV. Adverse pregnancy outcomes were similar in both groups.

**Figure:**
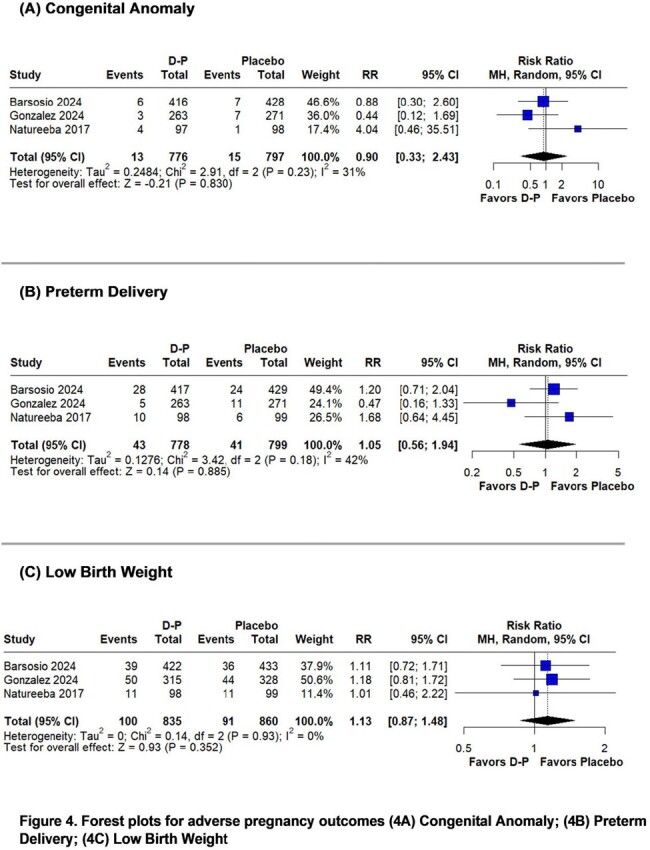

**Disclosures:**

**All Authors**: No reported disclosures

